# Case Report: The intersection of psychiatry and medicine: diagnostic and ethical insights from case studies

**DOI:** 10.3389/fpsyt.2025.1576179

**Published:** 2025-04-22

**Authors:** Francesco Monaco, Annarita Vignapiano, Martina D’Angelo, Fabiola Raffone, Valeria Di Stefano, Antonella Boccia, Anna Longobardi, Benedetta Di Gruttola, Michele Fornaro, Giulio Corrivetti, Iolanda Martino, Luca Steardo, Luca Steardo

**Affiliations:** 1 Department of Mental Health, Azienda Sanitaria Locale (ASL) Salerno, Salerno, Italy; 2 European Biomedical Research Institute of Salerno (EBRIS), Salerno, Italy; 3 Psychiatric Unit, Department of Health Sciences, University Magna Graecia of Catanzaro, Catanzaro, Italy; 4 Department of Mental Health, Azienda Sanitaria Locale (ASL) Napoli 1 Centro, Napoli, Italy; 5 Section of Psychiatry, Department of Neuroscience, Reproductive Science, and Dentistry, Federico II University of Naples, Naples, Italy; 6 Department of Medical Sciences, Institute of Neurology, University “Magna Graecia” of Catanzaro, Catanzaro, Italy; 7 Department of Physiology and Pharmacology “Vittorio Erspamer”, Sapienza University of Rome, Rome, Italy; 8 Department of Clinical Psychology, University Giustino Fortunato, Benevento, Italy

**Keywords:** psychosomatic disorders, somatopsychic conditions, complex systemic disorders, diagnostic challenges, medicine-psychiatry interface, ethical implications

## Abstract

The intersection of psychiatry and medicine presents unique diagnostic and ethical challenges, particularly for conditions involving significant brain-body interactions, such as psychosomatic, somatopsychic, and complex systemic disorders. This article explores the historical and contemporary issues in diagnosing such conditions, emphasizing the fragmentation of medical and psychiatric knowledge, biases in clinical guidelines, and the mismanagement of complex illnesses. Diagnostic errors often arise from insufficient integration between general medicine and psychiatry, compounded by the reliance on population-based guidelines that neglect individual patient needs. Misclassification of conditions like myalgic encephalomyelitis/chronic fatigue syndrome (ME/CFS), Lyme disease, and fibromyalgia as psychosomatic or psychogenic has led to stigmatization and delayed care. While these conditions are referenced as emblematic examples of misclassified and poorly understood disorders, the five clinical cases discussed in this article do not directly illustrate these diseases. Instead, they exemplify shared diagnostic and ethical dilemmas at the medicine–psychiatry interface, including uncertainty, fragmentation, and the risk of epistemic injustice. The article critically examines terms like medically unexplained symptoms and functional disorders, highlighting their limitations and potential for misuse. Case examples underscore the consequences of diagnostic inaccuracies and the urgent need for improved approaches. Ethical considerations are also explored, emphasizing respecting patient experiences, promoting individualized care, and acknowledging the inherent uncertainties in medical diagnosis. Advances in technologies such as brain imaging and molecular diagnostics offer hope for bridging the gap between psychiatry and medicine, enabling more accurate assessments and better patient outcomes. The article concludes by advocating comprehensive training at the medicine-psychiatry interface and a patient-centered approach that integrates clinical observation, research insights, and a nuanced understanding of mind-body dynamics.

## Challenges in diagnosing complex conditions in the medicine-psychiatry interface

Diagnosing conditions that involve both general medical and psychiatric aspects presents significant challenges for many physicians, often resulting in errors that adversely affect patients (1). Historically, poorly understood illnesses have frequently been attributed to psychiatric causes until their pathophysiology was better elucidated, as exemplified by the historical attribution of peptic ulcer disease primarily to psychological factors prior to the recognition of Helicobacter as one of its principal causes, alongside NSAID use (2). In recent years, the focus on standardized guidelines and computerized algorithms, combined with increasing sub-specialization, has led to fragmented knowledge and limitations in adequately assessing complex conditions. Studies indicate clinicians often interrupt patients within seconds of the consultation, hindering the thorough collection of essential information (3). These challenges stem from several factors: insufficient training in either medicine or psychiatry, gaps between clinical experience and research, difficulties in applying group-based research findings to individual patients, and the inappropriate use of guidelines. For instance, internal medicine specialists, who often draft clinical guidelines, typically have limited exposure to psychiatry. Similarly, not all psychiatrists and mental health professionals maintain up-to-date knowledge of general medicine. This fragmentation leaves many conditions at the interface of psychiatry and medicine inadequately addressed (4).

Another issue involves translating research into clinical practice. While researchers generate statistical data on groups of patients, applying these findings to individual cases remains problematic (5). Greater integration of clinical observations with basic research could improve outcomes. However, clinical guidelines are often developed by experts with limited direct patient care experience, as exemplified by the diagnostic criteria for Lyme disease, predominantly defined by researchers rather than community physicians with practical expertise (6).

This emphasis on population-based standards of care undermines the personalized management of complex diseases, particularly those involving brain-body interactions (7). Rigid adherence to guidelines without exercising sound clinical judgment risks compromising care quality, leaving patients with challenging conditions unsupported and exacerbating their sense of abandonment.

## Consequences of diagnostic errors in complex and misunderstood conditions

Diagnosing complex brain-body conditions presents significant challenges for healthcare providers, insurers, and patients alike. Many patients report consulting multiple physicians before receiving an accurate diagnosis. For instance, a survey of over 12,000 individuals with Lyme disease revealed that patients typically saw an average of five doctors prior to being correctly diagnosed (8). The constraints of healthcare reimbursement systems often limit the time available for comprehensive assessments, prompting physicians to order excessive tests. This approach strains financial resources, as these patients frequently fall outside conventional diagnostic frameworks, leading to costly evaluations with limited efficacy (9). Delays in diagnosis also result in increased costs due to disability, reduced productivity, and caregiving burdens, impacting insurers, government healthcare programs, employers, and families (10). Additionally, patients with complex or chronic conditions often face significant out-of-pocket expenses, contributing to medical bankruptcies among both insured and uninsured individuals (11).

These diagnostic difficulties are further compounded by biases in how symptoms are perceived and addressed. Female patients, for example, are disproportionately labeled with incorrect psychosomatic diagnoses, reflecting gender biases and inadequate understanding of female-specific physiological responses (12). Many women report being dismissed by healthcare providers, such as receiving anxiety medications during a heart attack or having autoimmune diseases mischaracterized as chronic complaints. Conditions like chronic fatigue syndrome and fibromyalgia are often dismissed as “hysteria” or “imaginary,” leaving patients stigmatized and misunderstood (13).

High-profile cases highlight the severe consequences of diagnostic errors. In one instance, a young woman with Lyme disease was cleared of psychological issues by two psychiatrists, but her pediatrician refused to accept these findings and subjected her to harmful practices. Another case in the UK involved a woman misdiagnosed with somatization disorder, resulting in a 20-year delay before identifying the true cause of her symptoms. Similarly, medical literature recounts the story of a woman misdiagnosed with somatic symptom disorder, only to be diagnosed with multiple myeloma after a proper evaluation (14).

Emerging research reveals connections between infections and psychiatric symptoms, suggesting that some cases previously attributed to psychological causes may instead have pathobiological origins (15). For example, a study in the Dutch General Practice Registry found that patients diagnosed with somatoform disorders had higher infection rates prior to their diagnosis. However, conclusions often misrepresent this as psychological distress causing physical symptoms, rather than recognizing the inverse relationship.

Patients with conditions that are difficult to diagnose, such as myalgic encephalomyelitis/chronic fatigue syndrome, fibromyalgia, Lyme disease, and postural orthostatic tachycardia syndrome (POTS) are frequently overlooked due to the lack of visible signs (16, 17). They are often told their symptoms are imaginary or self-inflicted, leading to feelings of abandonment and mistrust in the healthcare system. These experiences heighten the risk of mental health struggles, including suicidal ideation and attempts, underscoring the urgent need for better diagnostic approaches.

## Limitations and biases in medical guidelines: implications for patient care

All medical guidelines come with inherent limitations and disclaimers, emphasizing the need for individualized clinical judgment (18). Guidelines often rely on randomized controlled trials, which have two key limitations: (1) Once a certain level of knowledge is attained, it becomes unethical to continue placebo-controlled studies; (2) Research findings on groups may not always be applicable to individual patients.

These constraints highlight those guidelines, while valuable, cannot be universally applied.

Flaws in guidelines often stem from biased or inadequate research. When well-meaning physicians follow these flawed recommendations, patient care can suffer (19). Examples include guidelines addressing myalgic encephalomyelitis/chronic fatigue syndrome (ME/CFS), Lyme disease, and medically unexplained symptoms (MUS) (20). A lack of understanding of ME/CFS, even following the Institute of Medicine’s report, has left many patients feeling ignored or mistreated (21, 22). Over 80% of ME/CFS patients go undiagnosed, with 65% spending more than a year seeking an accurate diagnosis (23). Flawed recommendations, such as those from the PACE trial, erroneously suggested ignoring symptoms and relying on cognitive behavioral therapy (CBT) and graded exercise therapy for recovery (24). These approaches were not supported by robust evidence, and subsequent reviews, including by Cochrane, deemed the research inadequate. Revised guidelines have since improved patient management.

Similarly, the Infectious Diseases Society of America (IDSA) guidelines for Lyme disease failed to address key aspects of brain-body interactions (25, 26). They relied heavily on flawed testing and dismissed late-stage symptoms as minor or unexplained, leading to widespread criticism for bias and lack of transparency. The Institute of Medicine’s report, Clinical Practice Guidelines We Can Trust, highlighted these guidelines as an example of untrustworthiness (27). Although revisions were attempted in 2019, the updates made minimal progress in addressing controversies.

Another problematic guideline, Medically Unexplained Symptoms (MUS) in Children and Young People, endorsed by the Royal College of Psychiatrists and the Pediatric Mental Health Association, exhibits biases that prioritize the interests of physicians or third parties over those of patients (28, 29). Despite the APA’s 2013 removal of MUS as a valid diagnostic concept in the DSM 5-TR, the guideline encourages clinicians to diagnose MUS based on subjective criteria, such as patient history of extensive investigations, physician frustration with the patient’s lack of improvement, or familial patterns. These subjective judgments risk perpetuating stigma and misdiagnosis, undermining patient care.

## Methods

This study presents 5 cases examples to illustrate the challenges in distinguishing psychosomatic, somatopsychic, and multisystem illnesses. To protect confidentiality, patient identities were anonymized, and written consent for publication was obtained. To support differential diagnoses, key brain-body diagnostic terms associated with ambiguity, controversy, misdiagnoses, and potential misuse are identified and analyzed. These terms were gathered from the first author’s clinical experience in consultation-liaison psychiatry, as well as through searches on PubMed, Google Scholar, and the author’s archives.

Definitions and discussions are grounded in formal diagnostic references, including the DSM-5-TR and the International Classification of Diseases (ICD). Some terms are referenced in one or both systems, while others exist outside these formal classifications. Diagnostic categories examined include mental health, mental illness, psychosomatic and somatopsychic disorders, multisystem illnesses, medical uncertainty, somatoform disorders, medically unexplained symptoms, functional and psychogenic disorders, compensation neurosis, psychogenic seizures, psychogenic pain and fatigue, delusional parasitosis, and bodily distress disorders. Relevant DSM-5 codes and their corresponding ICD classifications are provided.

The review emphasizes terms most susceptible to misuse and diagnostic inaccuracies, focusing on their implications for clinical practice. Articles defining and differentiating these conditions are critically assessed, with key conclusions drawn to aid clinicians in accurately navigating the complex interplay between psychosomatic, somatopsychic, and multisystem conditions, as well as addressing medical uncertainty. The selection of clinical cases presented in this article was not intended to offer a direct representation of specific conditions such as ME/CFS, Lyme disease, or fibromyalgia, which are referenced in the introduction as paradigmatic examples of misclassified or misunderstood illnesses. Rather, these cases were chosen to illustrate a set of shared challenges commonly encountered at the interface between psychiatry and general medicine, namely, diagnostic uncertainty, fragmentation of care, epistemic injustice, and the premature psychologization of physical symptoms. Although the selected cases vary nosologically ranging from recognized psychiatric disorders such as anorexia nervosa to functional neurological and gastrointestinal syndromes, they all exemplify clinical situations in which patients face inconsistent recognition of symptom legitimacy, diagnostic ambiguity, and difficulty accessing integrated, interdisciplinary care. These issues reflect the broader conceptual concerns discussed throughout the manuscript, particularly the limitations of current diagnostic frameworks and the ethical implications of labeling. By presenting cases across this diverse clinical spectrum, we aim to underscore the need for a more nuanced, integrative, and patient-centered diagnostic approach to complex disorders involving brain-body interactions.

### Case 1: anorexia nervosa subtype binge/purge and borderline personality disorder in an adolescent

This case report presents the complex clinical picture of a 17-year-old adolescent female diagnosed with Anorexia Nervosa (AN) binge/purge subtype and comorbid Borderline Personality Disorder (BPD). Her condition was complicated by recurrent seizure-like episodes and multiple Emergency Department (ED) visits, posing significant challenges in assessing disease severity and guiding treatment. The patient had a family history of hypothyroidism, anxiety, and depressive disorders, while her early development was reported as normal, including an uncomplicated pregnancy, breastfeeding for eight months, and appropriate psychomotor milestones, with menarche occurring at age 12.

Her clinical deterioration began in December 2022, marked by severe dietary restrictions alternating with binge eating and self-induced vomiting, leading to rapid weight loss and amenorrhea. She also experienced recurrent seizure-like episodes, despite normal EEG findings, which, combined with affective instability, irritability, and episodes of anger, resulted in frequent ED visits. Upon admission to the Residential Center for Eating Disorders “Mariconda” (ASL Salerno), the severity of her condition became evident. She displayed bradycardia, hypothermia, and dehydration, alongside clear signs of malnutrition, with laboratory tests confirming hypokalemia and other electrolyte imbalances. Despite inpatient care, she required additional ED visits due to persistent bradycardia, hypothermia, and dehydration, as well as further seizure-like episodes, underscoring the urgent need for intensive therapeutic intervention. The final diagnosis confirmed AN binge/purge subtype and BPD, with associated anxiety, specific phobias, and interpersonal difficulties.

A comprehensive diagnostic assessment was conducted to evaluate both her eating disorder and comorbid psychological symptoms. Standardized instruments, including the SCL-90, Eating Disorder Examination (EDE), Body Uneasiness Test (BUT), and Eating Disorder Inventory-3 (EDI-3), revealed a high level of general psychological distress, severely impaired eating behaviors, significant body dissatisfaction, and interpersonal difficulties. The assessment also incorporated physical parameters, confirming low body weight, bradycardia, hypothermia, and electrolyte imbalances. Beyond their clinical implications, the seizure-like episodes had a profound psychological impact, generating significant anxiety and fear, which interfered with her daily functioning and social relationships. The repeated ED visits contributed to feelings of failure and shame, further worsening her emotional state and self-esteem. The burden extended to her family, who experienced heightened anxiety due to the frequency and severity of these acute episodes, creating additional stress and challenges in managing daily life. The economic burden associated with repeated medical evaluations, hospitalizations, and specialized consultations added further strain to the healthcare system.

Her treatment plan required a multidisciplinary approach, including psychotherapy (individual and group), structured nutritional rehabilitation, and pharmacotherapy. Initially, she was prescribed Fluvoxamine, Delorazepam, and Talofen, with later adjustments introducing Fluvoxamine and Lurasidone. Given her clinical instability, managing her seizure-like episodes and preventing further ED visits became a priority, necessitating continuous monitoring of her vital signs and thorough evaluation of her physical condition.

This case highlights the complex interaction between AN and BPD in adolescence, emphasizing the need for an integrated medical-psychiatric approach. The unclear etiology of the seizure-like episodes added diagnostic complexity, requiring careful differential diagnosis to exclude organic neurological causes. The frequency of ED visits reflected her medical and psychological instability, demanding constant monitoring and a comprehensive intervention strategy. A positive treatment response was only achieved through an interdisciplinary collaboration between psychologists, nutritionists, and psychiatrists, reinforcing the importance of holistic care. Family involvement played a crucial role in reducing stress and improving treatment adherence. The challenges presented in this case underscore the necessity of early intervention and ongoing assessment of potential medical complications, such as cardiac arrhythmias due to electrolyte imbalances.

Managing patients with AN and BPD, particularly those with seizure-like episodes and frequent ED visits, requires a specialized multidisciplinary team with expertise in psychiatry, neurology, and nutrition. Close monitoring of both physical and psychological parameters is essential to prevent further medical complications and ensure clinical stabilization. A flexible therapeutic approach is critical to adapt to fluctuating symptoms and the evolving needs of both the patient and her family. Early intervention, coordinated communication among healthcare providers, and psychological support for family members are fundamental to long-term management, aiming to improve quality of life while reducing psychological distress and financial strain on the healthcare system.

### Case 2: a young woman with anorexia nervosa and prominent somatic symptoms

This case report details the clinical presentation, assessment, treatment, and outcome of a young woman diagnosed with anorexia nervosa (AN) with prominent somatic complaints, emphasizing the importance of a holistic approach to diagnosis and management. The patient, a 17-year-old female, was a middle school student at the onset of treatment. Her family history revealed hypothyroidism in both parents and a background of anxiety and depressive disorders. According to her parents, she experienced normal physiological development, including an uncomplicated pregnancy and delivery, breastfeeding for eight months, and appropriate psychomotor milestones, with menarche occurring at age 12.

Her clinical history revealed a diagnosis of AN at age 13, leading to multiple hospitalizations due to severe weight loss, followed by a period of residential treatment. Over time, she developed a constellation of persistent somatic complaints, including severe fatigue, impaired concentration, and memory, difficulty completing tasks, and a pervasive sense of malaise. These symptoms were consistently reported across multiple assessments and were supported by psychometric testing, which indicated a marked tendency toward somatization. During residential treatment, she required multiple emergency room visits due to episodes of acute agitation with partial loss of consciousness, further complicating her clinical course.

To gain a comprehensive understanding of her psychological state and its relationship to her AN, she underwent an extensive psychological assessment using standardized instruments, including the Minnesota Multiphasic Personality Inventory-2 (MMPI-2), the Symptom Checklist-90-Revised (SCL-90-R), the Eating Disorder Examination Questionnaire (EDE-Q), and the Eating Disorders Inventory-3 (EDI-3). The MMPI-2 provided a detailed psychological profile, revealing significant psychological distress, a pronounced tendency to somatize negative emotions, and notable difficulties with concentration and task completion. The SCL-90-R confirmed significant elevations across multiple subscales, indicating substantial anxiety, depression, hostility, and somatization, corroborating her subjective reports and objectively supporting the presence of comorbid conditions alongside AN. The EDE-Q and EDI-3 provided further insight into the severity and specific characteristics of her eating disorder, highlighting body dissatisfaction, dysfunctional eating behaviors, interpersonal difficulties, and low self-esteem. Collectively, these findings supported a diagnosis of AN with significant comorbid anxiety and depressive disorders, reinforcing the need for a comprehensive, integrated treatment plan that addressed both her eating disorder and psychological symptoms.

Given the complexity of her condition, treatment required a multidisciplinary approach that included individual and group psychotherapy, intensive nutritional rehabilitation, and pharmacotherapy. Initially, she was prescribed Sertraline and Olanzapine, with Fluvoxamine and Lurasidone added later. Managing her acute agitation and preventing further emergency room visits became a primary concern, necessitating close monitoring of her vital signs and careful assessment of her physical condition to ensure stability.

This case highlights the intricate relationship between psychological distress and somatic symptoms in AN, underscoring the importance of a holistic approach that integrates both physical and psychological aspects of care. The presence of prominent somatic symptoms, evident throughout her history and confirmed by psychometric testing, reinforces the necessity of a comprehensive evaluation, incorporating physical examinations, laboratory investigations to rule out organic causes, and detailed psychological assessments. Effective treatment strategies must extend beyond weight restoration, addressing the underlying psychological mechanisms that perpetuate both AN and its associated somatic manifestations.

This case underscores the critical need for an integrated, multidisciplinary approach that considers both somatic and psychological components in the management of AN. Early intervention and a comprehensive treatment strategy, incorporating nutritional rehabilitation, psychotherapy, and when necessary, pharmacotherapy, are crucial for improving outcomes. Further research is warranted to better understand the pathophysiological mechanisms linking AN and its diverse somatic manifestations and to refine treatment strategies for this complex and vulnerable patient population.

### Case 3: anorexia nervosa and comorbid obsessive-compulsive disorder

A.E., a 16-year-old student, has a complex clinical history marked by Anorexia Nervosa and comorbid Obsessive-Compulsive Disorder (OCD), highlighting the intricate relationship between psychiatric and somatic manifestations. Born after a full-term, uncomplicated pregnancy, her early development was within the normal range, with no significant delays. However, by adolescence, she exhibited a progressive decline in health, leading to hospital admissions for weight loss and malnutrition, with a BMI of 15.3 at her first neuropsychiatric hospitalization in 2021. Following multiple inpatient treatments, including a residential program at a specialized eating disorder facility, she continued to struggle with the somatization of emotional distress, as evidenced by psychodiagnostics assessments. Her MMPI-2 results suggest a tendency to amplify health concerns and somatic symptoms, experiencing fatigue, cognitive difficulties, and impaired daily functioning. While maintaining a certain level of self-confidence, she displays impulsivity, irritability, and episodes of verbal or physical aggression, pointing to emotional dysregulation. The SCL-90 revealed severe psychological distress, with high scores in phobic anxiety, psychoticism, hostility, and depression, reinforcing the hypothesis of a strong psychosomatic interplay. Additional scales, such as the EDS, CIA, BUT, and EDE-Q, indicated significant body image concerns, excessive self-monitoring, and risk of developing compulsive physical activity patterns, reinforcing the somatic expression of underlying psychiatric distress. On August 19, 2024, Alessandra was brought to the Emergency Department (ED) in a state of agitation, coinciding with her admission to another residential eating disorder treatment facility. Despite her distress and resistance, no acute psychiatric conditions were noted, and her thought processes remained logically structured, with mood stability and no psychotic symptoms or suicidal ideation. Her pharmacological treatment has included Sertraline, Olanzapine, Alprazolam (as needed), Lurasidone, and Fluvoxamine, reflecting the complexity of managing both mood instability and obsessive-compulsive features alongside her restrictive eating behaviors. Clinically, Alessandra displays a marked tendency toward externalizing blame, often feeling misunderstood, unfairly punished, or surveilled by others, suggestive of paranoid ideation. She also exhibits social anxiety, emotional detachment, and pervasive distrust, coupled with restlessness, cognitive acceleration, and difficulty regulating affective states, leading to frequent emotional outbursts. Her overall mood remains depressed and dysphoric, dominated by feelings of inadequacy, dissatisfaction, and chronic unhappiness. This case underscores the bidirectional relationship between psychiatric conditions and their somatic manifestations, illustrating how an eating disorder not only alters physical health but also exacerbates underlying psychopathological dimensions. Alessandra’s presentation highlights the need for integrated psychiatric-medical approaches, as rigid diagnostic categories often fail to capture the multifaceted nature of psychosomatic conditions, where emotional distress is not only expressed through the body but also perpetuated by physiological deterioration.

### Case 4: functional neurological disorder in a young woman with persistent sensorimotor symptoms

This case presents the complex diagnostic and therapeutic challenges of a 19-year-old female university student, diagnosed with Functional Neurological Disorder (FND) after an extensive medical workup failed to identify an organic cause for her progressively disabling sensorimotor symptoms. The patient, an academically high-achieving and socially engaged individual, presented with episodic limb weakness, intermittent non-epileptic seizures, gait disturbances, and sensory deficits, leading to multiple Emergency Department (ED) visits and hospital admissions. Despite exhaustive neurological and medical evaluations, including MRI, EEG, lumbar puncture, and autoimmune panels, no structural, inflammatory, or metabolic abnormalities were identified.

The onset of symptoms occurred six months before her first ED visit, initially manifesting as unilateral leg weakness and a tingling sensation in the right arm. Over time, her symptoms progressed to intermittent paralysis, involuntary jerking movements, and dissociative episodes, significantly impairing her mobility and academic performance. The patient’s episodes often occurred in stressful situations but could also arise unexpectedly, lasting from minutes to hours. These episodes were frequently accompanied by emotional distress, hyperventilation, and a subjective sense of detachment from her body. Notably, neurological examinations revealed inconsistencies, including Hoover’s sign, a hallmark of functional motor weakness, and variable sensory deficits that did not follow anatomical patterns.

Her personal history included a childhood marked by high parental expectations and perfectionistic tendencies, though she denied significant past psychiatric illnesses. However, she had a history of anxiety, panic attacks, and frequent somatic complaints during adolescence, including gastrointestinal distress and chronic fatigue, often dismissed as stress-related. Her family history was significant for migraines, fibromyalgia, and generalized anxiety disorder, but no known neurological conditions.

During hospital admissions, a psychiatric consultation was requested due to the absence of identifiable organic pathology, leading to a diagnosis of Functional Neurological Disorder (FND), subtype motor and sensorimotor dysfunction, with probable dissociative features. The diagnosis was explained to the patient and her family using a biopsychosocial model, emphasizing the real nature of her symptoms, their reversibility, and the role of maladaptive brain processing rather than conscious fabrication. However, the initial acceptance of this diagnosis was challenging, as the patient and her family perceived the symptoms as evidence of an undiagnosed neurological disease, reflecting the stigma and misunderstandings often associated with functional disorders.

A multidisciplinary treatment plan was implemented, combining neurology, psychiatry, physiotherapy, and cognitive-behavioral therapy (CBT). The key treatment components included education on FND, graded physical rehabilitation to restore movement confidence, and psychotherapy targeting stressors, maladaptive beliefs, and emotion regulation difficulties. Pharmacotherapy with low-dose Sertraline was introduced to manage coexisting anxiety symptoms, while benzodiazepines were avoided due to their potential to reinforce symptom expression.

Over the course of six months, the patient showed gradual improvements, with a reduction in seizure-like episodes and improved motor function. However, setbacks occurred, particularly during academic stressors, requiring ongoing therapy and structured symptom management strategies. By the one-year follow-up, the patient had regained significant functionality, though occasional transient episodes of limb weakness and dissociative symptoms persisted under stress.

This case underscores the diagnostic and ethical complexities of FND, where the absence of structural pathology often leads to skepticism, delayed diagnosis, and misattribution to malingering or psychiatric illness. It highlights the importance of early diagnosis, patient-centered communication, and an integrative treatment approach that acknowledges the interplay between neurological dysfunction, psychological distress, and sensorimotor processing abnormalities. Furthermore, it reflects the ongoing challenges in bridging the gap between psychiatry and neurology, advocating for enhanced physician education on FND to reduce diagnostic bias and improve patient outcomes.

### Case 5: a young man with severe gastrointestinal symptoms and underlying psychosomatic disorder

This case highlights the complex interplay between psychological distress and somatic symptoms in a 22-year-old male university student diagnosed with Severe Functional Gastrointestinal Disorder (FGID) with a psychophysiological component, emphasizing the challenges in differentiating primary organic pathology from psychogenic influences.

The patient initially presented with persistent abdominal pain, nausea, bloating, and alternating diarrhea and constipation, which had progressively worsened over the past two years. His symptoms significantly impaired his daily life, leading to severe weight loss, dietary restrictions, and avoidance of social situations due to a fear of unpredictable gastrointestinal distress. Despite multiple consultations with gastroenterologists and internal medicine specialists, extensive medical workups—including endoscopies, colonoscopies, abdominal imaging, and stool and blood tests—failed to reveal any structural, infectious, or inflammatory causes.

During repeated Emergency Department (ED) visits, the patient frequently reported excruciating abdominal pain and an inability to tolerate food, yet clinical examinations consistently revealed no signs of acute pathology, normal laboratory markers, and no evidence of intestinal obstruction or inflammation. Due to the chronicity and persistence of symptoms despite normal investigations, he was referred for a psychiatric and psychological evaluation to assess the possibility of an underlying psychosomatic disorder.

A detailed psychosocial history revealed a pattern of high anxiety, excessive health concerns, and a history of childhood stressors, including perfectionistic tendencies, high parental expectations, and a fear of failure. The patient described early-life gastrointestinal complaints, which had intensified during high-stress periods, particularly before exams or social engagements. He admitted to catastrophic thinking about his symptoms, often fearing he had an undiagnosed life-threatening illness despite multiple reassurances. Standardized psychometric assessments, including the SCL-90, Hospital Anxiety and Depression Scale (HADS), and Patient Health Questionnaire (PHQ-15), indicated high levels of somatic symptom amplification, significant anxiety, and mild depressive features, reinforcing the hypothesis of a psychosomatic contribution to his condition.

Given the absence of an organic explanation for his severe gastrointestinal distress, a diagnosis of Somatic Symptom Disorder with predominant gastrointestinal manifestations (formerly known as Psychosomatic Gastrointestinal Disorder) was established. The patient’s symptoms were conceptualized within a biopsychosocial model, emphasizing heightened gut-brain axis dysregulation, visceral hypersensitivity, and autonomic nervous system hyperactivity in response to psychological stress. The patient initially resisted the diagnosis, interpreting it as a dismissal of his symptoms, a reaction commonly seen in patients with psychophysiological disorders due to the stigma surrounding psychosomatic conditions.

A multidisciplinary treatment approach was initiated, including psychoeducation on the gut-brain connection, cognitive-behavioral therapy (CBT) to address health-related anxiety and avoidance behaviors, and gut-directed hypnotherapy to modulate visceral pain perception. A trial of low-dose tricyclic antidepressants (Amitriptyline 10 mg) was introduced to reduce neuropathic pain sensitivity and visceral hyperalgesia, while serotonergic agents were cautiously adjusted to avoid exacerbating gastrointestinal motility symptoms. Nutritional counseling was integrated to normalize dietary patterns and reduce restrictive eating behaviors linked to symptom anxiety.

Over six months, the patient experienced a gradual reduction in symptom severity, regained lost weight, and began to reintegrate into social and academic life. However, relapses occurred during high-stress periods, reinforcing the importance of ongoing psychological support and stress management strategies. By the one-year follow-up, he had achieved significant functional improvement, though he remained vulnerable to episodic symptom flare-ups in response to emotional distress or environmental triggers.

This case illustrates the profound impact of psychosomatic disorders on physical health and the need for a paradigm shift in medical practice, where functional symptoms are recognized as real and treated within an integrative framework rather than dismissed as purely psychiatric. The diagnostic challenge in distinguishing psychosomatic disorders from organic pathology underscores the importance of a multidisciplinary approach, where both medical and psychological components are addressed without invalidating the patient’s experience. The case also highlights the need for greater physician education on psychophysiological disorders, emphasizing the role of neurobiological mechanisms underlying psychosomatic symptoms to improve diagnostic accuracy and patient outcomes.

## Discussion of case presentations

The presented case series underscores the intricate diagnostic challenges and ethical considerations at the intersection of psychiatry and medicine, particularly in conditions where psychosomatic and functional manifestations prevail. Each case illustrates the profound impact of psychological distress on physical health, revealing how disorders such as anorexia nervosa, functional neurological disorders, and severe functional gastrointestinal disorders blur the lines between psychiatric and medical domains. These cases emphasize the necessity of an integrated, multidisciplinary approach that embraces both physical and psychological dimensions of care, countering the risks of misdiagnosis and stigma often associated with somatic symptom disorders. The nuanced interplay between emotional and somatic symptoms demands a biopsychosocial model of understanding, one that respects patient experiences and acknowledges the limitations of current diagnostic frameworks. Although the bio-psycho-social model remains a valuable conceptual framework for understanding complex disorders involving brain-body interactions, its implementation in real-world clinical practice has been limited. Structural fragmentation between specialties, time constraints during medical consultations, and insufficient integration of behavioral health into general care all contribute to the gap between theoretical ideals and actual practice. As highlighted by Presseau et al. (30) and Epstein et al. (31), his discrepancy reflects broader challenges in translating behavioral and health psychology research into consistent, reproducible clinical strategies (30, 31). Enhanced training for clinicians at this interface, alongside patient-centered communication, is pivotal for advancing diagnostic accuracy and therapeutic outcomes. Ultimately, these cases advocate for a paradigm shift toward holistic, individualized care, where the complexities of mind-body interactions are navigated with empathy and scientific rigor.

## Discussion

### Defining mental illness: conceptual frameworks, pathophysiology, and integrative perspectives

Mental illness also referred to as psychiatric illness or mental disorder, is defined by the DSM-5-TR as a clinically significant disturbance in cognition, emotional regulation, or behavior, reflecting dysfunction in psychological, biological, or developmental processes (32). Similarly, the ICD characterizes mental disorder as a clinically recognizable set of symptoms or behaviors typically associated with distress and functional impairment. A more functional approach, inspired by the U.S. Surgeon General’s Mental Health Report, conceptualizes mental illness as a disruption of adaptive capabilities, affecting productivity and sense of purpose, interpersonal relationships and emotional resilience, as well as enjoyment of life and adaptability to change (33). These varying definitions highlight the multifaceted nature of psychiatric disorders, integrating neuroscientific, psychological, and sociocultural perspectives.

While the DSM-5-TR provides categorical classifications, it does not elucidate the neurobiological and pathophysiological mechanisms underlying psychiatric disorders (34). Mental illness arises from dynamic interactions among genetic, environmental, neurodevelopmental, and immune-inflammatory factors, leading to functional and structural alterations in neural circuits (35). These disruptions manifest across three major neurophysiological domains: cognitive dysfunction due to impaired prefrontal networks affecting executive function, attention, and cognitive flexibility; emotional dysregulation characterized by amygdala hyperactivity, impaired emotional processing, and maladaptive reward system function; and autonomic and vegetative dysfunction involving the brainstem and hypothalamic dysregulation, leading to altered arousal, sleep-wake regulation, and stress responses (36, 37).

From a systems perspective, mental health represents a dynamic equilibrium between protective and risk factors, while mental illness reflects a disruption in this balance, triggering a pathological cascade (38). This pathophysiological sequence often involves genetic vulnerabilities such as neurotransmitter gene polymorphisms and epigenetic modifications, environmental stressors including trauma, chronic inflammation, and early-life adversity, as well as neuroimmune and inflammatory dysregulation contributing to oxidative stress, neuroinflammation, and dysregulated neurotransmission. These cumulative biological and environmental factors drive the development of distinct symptom clusters and syndromes as categorized in the DSM-5-TR (39). Notably, delayed diagnosis and inadequate intervention exacerbate neuroprogression, increasing disease chronicity and functional decline (40).

### Establishing diagnostic precision in complex conditions

When encountering complex conditions that remain insufficiently explored or poorly supported by standard clinical investigations, the clarity of diagnostic definitions becomes paramount. As Socrates observed, “The beginning of wisdom is the definition of terms” (41). In cases where mind-body interactions are central, a precise conceptual framework is essential for differentiating psychiatric, functional, and systemic disorders (42). This requires explicit definitions of key terms, including mental illness, psychosomatic and somatopsychic disorders, multisystem conditions, and medical uncertainty, as classified in the DSM-5-TR and ICD (43, 44).

The Diagnostic and Statistical Manual of Mental Disorders (DSM), first introduced in 1952 by the American Psychiatric Association, evolved from the 1948 International Lists of Diseases and Causes of Death, establishing standardized psychiatric diagnoses for clinical applicability (45). Over time, both the DSM and the International Classification of Diseases (ICD) have developed independently yet in parallel, integrating advances in psychiatric and medical classifications (46). While ICD-10 remains widely used, the transition to ICD-11 introduces refined nosological criteria, enhancing diagnostic specificity and aligning psychiatric conditions with broader medical frameworks (43).

Establishing this structured diagnostic foundation enables clinicians to navigate the complexities of differential diagnosis with greater accuracy, reducing misclassification, diagnostic uncertainty, and biases in complex clinical cases. A rigorous, standardized approach fosters greater precision in assessment and management, ultimately improving patient outcomes in conditions at the intersection of psychiatry and medicine (47).

### Psychosomatic and somatopsychic disorders: definitions, mechanisms, and gaps in classification

The term psychosomatic is not explicitly included in the DSM-5-TR, as this classification system focuses on symptoms and syndromes rather than causality (48). Similarly, while the ICD addresses causality in some diagnostic categories, it does not specifically define psychosomatic conditions. This creates significant gaps in the standardization of definitions and classifications for these disorders (49).

Psychosomatic disorders refer to physical illnesses caused or exacerbated by mental stress and emotional distress (50). As scientific understanding advances, the list of conditions attributed solely to psychosomatic origins has diminished. For example, tuberculosis, hypertension, and stomach ulcers were once thought to have psychosomatic etiology. However, many illnesses, such as heart disease, irritable bowel syndrome (IBS), and muscle tension, are now recognized as having psychosomatic contributors, where stress exacerbates symptoms (51, 52).

Stress increases allostatic load, the cumulative wear and tear on the body leading to physiological changes, such as:

- Shifting the autonomic nervous system balance toward sympathetic dominance.
- Altering hypothalamic-pituitary-adrenal (HPA) axis activity.
- Raising blood pressure, heart rate, and respiratory rate.
- Elevating blood glucose levels.
- Enhancing blood flow to skeletal muscles.
- Promoting inflammation.
- Reducing regenerative and digestive activities.
- Decreasing blood flow to the prefrontal cortex under high stress (53).

Acute stress is generally manageable, but chronic, unresolved stress can lead to significant adverse effects, particularly in genetically or biologically susceptible individuals. These changes may result in psychosomatic symptoms and disorders (54).

For instance, psychosomatic cardiovascular disease in a vulnerable individual involves chronic activation of the HPA axis and sympathetic nervous system (55, 56). This reduces vagal tone, elevates catecholamine levels, and increases proinflammatory cytokines, leading to endothelial damage, plaque buildup, vascular occlusion, and higher risks of acute coronary syndromes and stroke (57).

Another example is irritable bowel syndrome (IBS), where chronic stress disrupts parasympathetic activity, leading to spastic peristalsis, diarrhea, constipation, or bowel urgency (58). Stress-related exacerbation can be compounded by dietary triggers, such as gluten or lactose intolerance (59).

In contrast, somatopsychic disorders describe mental illnesses caused or exacerbated by physical conditions. Unlike psychosomatic disorders, the range of somatic contributors to psychiatric symptoms continues to expand with ongoing research. Conditions such as endocrine dysfunctions, tumors, autoimmune diseases, and infections are increasingly recognized as triggers for psychiatric symptoms. For example, viral, venereal, and vector-borne infections often provoke immune-mediated psychiatric symptoms, as supported by numerous peer-reviewed studies (15). However, neither the DSM-5-TR nor the ICD explicitly defines somatopsychic disorders, leaving significant gaps in classification and standardization.

### Rethinking diagnostic models: integrating brain-body interactions for greater precision

The challenge of medical uncertainty is particularly evident in multisystem disorders, where complex interactions between neurological, immune, endocrine, and metabolic pathways blur the boundaries between psychiatric and somatic conditions. Current classification systems, such as the DSM-5-TR and ICD, rely on categorical rather than mechanistic frameworks, leading to inconsistencies in nomenclature, nosological gaps, and variability in clinical practice. This limitation is exemplified by the lack of clear distinctions between somatopsychic disorders, psychosomatic syndromes, and complex systemic diseases, resulting in fragmented care, misdiagnosis, and an overreliance on exclusion-based diagnostics. Emerging research in neurobiology, psychoneuroimmunology, and computational medicine challenges traditional dualistic perspectives, emphasizing the need for an integrative, multidimensional model that incorporates neurobiological, immunological, and psychosocial determinants (60). Discrepancies between the DSM-5-TR and ICD-11, particularly regarding conditions like bodily distress disorder (BDD), further underscore the need for conceptual clarity, as these differences influence clinical decision-making, treatment approaches, and healthcare policy (61). Advances in neuroimaging, biomarker research, and computational psychiatry hold promise for refining diagnostic classifications, enabling greater precision in delineating psychiatric and somatic pathologies (62). Future revisions of diagnostic frameworks must integrate these evolving insights to enhance their clinical relevance and scientific robustness (63). A paradigm shift is a necessary one that moves beyond rigid categorical distinctions, acknowledges the bidirectional interplay between mental and physical health, fosters interdisciplinary collaboration, and prioritizes individualized, evidence-based interventions (64). Such an approach is critical to reducing diagnostic ambiguity, improving clinical outcomes, and fostering a more nuanced understanding of the brain-body continuum in modern medicine (65).

### Differential diagnosis and the role of neuroimaging in disorders at the medicine-psychiatry interface

Differential diagnosis in conditions at the intersection of psychiatry and general medicine requires a thorough clinical, laboratory, and instrumental evaluation (66). In the cases presented, various alternative conditions were considered before reaching a definitive diagnosis. Neurological, endocrine, and immunological disorders often overlap with clinical presentations that have a strong psychosomatic component, making a multimodal assessment essential.

In the case of anorexia nervosa with seizure-like episodes, it was crucial to distinguish between generalized seizures, temporal lobe epilepsy, neurocardiogenic syncope, and functional neurological disorder (FND). Electroencephalography (EEG) was used to rule out an epileptic origin of the motor manifestations, while cardiological evaluation excluded anomalies suggestive of syncopal episodes. However, the current limitations of standard EEG in detecting deep epileptiform activity highlight the need for more advanced assessments, such as prolonged video-EEG monitoring or functional magnetic resonance imaging (fMRI), to identify functional alterations not visible with conventional imaging techniques.

In the case of the patient with functional neurological disorder (FND) and sensorimotor symptoms, organic neurological conditions such as multiple sclerosis, autoimmune neuropathies (e.g., Guillain-Barré syndrome), metabolic myopathies, and cerebrovascular diseases were excluded through brain and spinal MRI with contrast, cerebrospinal fluid analysis, and electrophysiological studies. The presence of positive clinical signs specific to FND, such as Hoover’s sign and symptom variability across repeated examinations, was crucial in establishing the diagnosis.

Regarding the patient with severe gastrointestinal symptoms, the differential diagnosis included inflammatory bowel diseases, gastrointestinal motility disorders, mast cell activation syndrome, and visceral hypersensitivity. Endoscopic examinations, celiac disease testing, and abdominal imaging ruled out a structural cause, supporting the hypothesis of a dysfunction in the gut-brain axis.

### FThe role of neuroimaging in differentiating functional conditions from structural alterations

Neuroimaging has become increasingly important in distinguishing functional neurological disorders from organic alterations (67). Studies using fMRI and PET have identified distinctive patterns in FND patients, suggesting the involvement of motor control circuits and limbic areas in symptom generation (68). In eating disorders, particularly anorexia nervosa, recent research has demonstrated abnormalities in the connectivity of the prefrontal cortex and insula, areas involved in appetite regulation, compulsive behavior, and body perception (69). Moreover, magnetic resonance spectroscopy (MRS) has revealed alterations in neurotransmitter levels, such as glutamate and GABA, suggesting potential biomarkers for monitoring treatment response (70).

Integrating these techniques into clinical practice could enhance diagnostic accuracy and provide tools to assess the effectiveness of therapeutic strategies, particularly in cases where the boundary between functional and organic etiology remains unclear.

### Addressing challenges in diagnosis, ethics, and individualized care

Identifying the sequential cause of a patient’s illness improves the potential for accurate diagnosis, effective treatment, and better outcomes. Although this work highlights the critical issues and potential risks associated with a rigid application of clinical guidelines in complex cases, it is equally important to acknowledge the recent progress made toward a more integrated and patient-centered approach. The updated NICE guidelines for ME/CFS, for instance, represent a significant paradigm shift, moving away from potentially harmful treatments such as graded exercise therapy and emphasizing the importance of validating patient experiences (71). Moreover, there is growing awareness of diagnostic biases particularly those related to gender and increasing recognition of the need to involve multidisciplinary teams in the development of clinical recommendations (19, 72). The evolution of international diagnostic classifications, such as the transition from ICD-10 to ICD-11, further reflects a commitment to greater nosological precision and improved integration between psychiatry and general medicine (43). Enhanced understanding can also help prevent errors by third parties, reducing misallocation of financial resources and regulatory efforts. However, many diagnostic terms remain prone to misuse and abuse. These include outdated concepts such as somatoform disorders and medically unexplained symptoms, as well as contentious diagnoses like bodily distress disorder and somatic symptom disorder (73).

Determining whether emotional distress causes somatic symptoms, somatic symptoms cause emotional distress, or a multisystem disorder underlies both can be complex. True medical uncertainty is often influenced by examining the physician’s knowledge and experience (74). These dynamic underscores the interplay of psychosomatic, somatopsychic, and multisystem illnesses, as well as the inherent challenges in distinguishing them (49).

As demonstrated in clinical cases, complex illnesses presenting later in life with numerous physical and mental symptoms are more likely to involve immune-mediated, multisystem conditions than psychosomatic disorders, which typically begin in childhood (50). Mislabeling such conditions can lead to delayed or inappropriate care.

The term “excessive” is central to the diagnoses of bodily distress disorder (BDD) and somatic symptom disorder (SSD) (75). Both rely on subjective evaluations of whether patients’ responses to symptoms are disproportionate. However, these assessments may be influenced by the limits of the clinical examination, physician bias, or external pressures, such as insurance company objectives. For example, the IDSA Lyme disease guidelines dismissed patients’ chronic symptoms as minor, yet NIH research found such patients experience pain and fatigue comparable to those with post-surgical pain or multiple sclerosis. Without objective criteria for “excessive,” these labels risk unfairly dismissing patients’ experiences.

Fatigue, the second most common complaint in primary care after chest pain, is often associated with proinflammatory states triggered by infections, cancer, or chronic illnesses. There is no evidence that fatigue in conditions like ME/CFS or Lyme disease is psychogenic. Instead, fatigue may cause mental distress, appearing earlier in disease progression, with depression typically arising later (76). While fatigue can accompany major depression, psychodynamic explanations for psychogenic fatigue lack support (77).

#### Ethical concerns

Respecting patients, maintaining the integrity of the physician-patient relationship, and prioritizing individualized care are ethical imperatives. However, pressures from third-party guidelines, misapplied research, and financial constraints threaten these principles. Diagnoses like bodily distress disorder may prioritize third-party interests or physician convenience over patient needs, dismissing significant symptoms and limiting thorough evaluations (78). Diagnoses should reflect patient-centered care, not serve to relieve the clinician’s distress or justify flawed systems. Furthermore, recent studies have highlighted the growing phenomenon of defensive psychiatry, in which clinicians may adopt overly cautious or risk-averse diagnostic strategies such as assigning broad psychiatric labels or avoiding complex differential diagnoses to protect themselves from legal liability or institutional scrutiny. While these practices may reduce perceived clinical risk, they often come at the cost of patient-centered care, leading to overdiagnosis, stigmatization, and reduced trust in the therapeutic relationship (79–81).

### Advancing the diagnosis and treatment of somatic symptom disorders

The revised conceptualization of somatic symptom disorders (SSDs) carries critical clinical and research implications, necessitating a more integrated diagnostic approach that incorporates medical, psychological, and neurobiological dimensions (82). The frequent misclassification of SSDs as purely psychiatric conditions highlights the need to avoid premature psychologization, particularly in cases where systemic, inflammatory, or neuroimmune mechanisms contribute to symptom persistence (64). Given the high prevalence of SSDs in both primary care and specialty settings, improving physician training is essential to enhance diagnostic accuracy, reduce clinician bias, and foster interdisciplinary collaboration across psychiatry, neurology, immunology, and internal medicine (65). The heterogeneous presentation of SSDs, including functional neurological symptoms, autonomic dysregulation, and stress-related somatic reactivity, underscores the necessity of a transdisciplinary framework that bridges these specialties. Future research should focus on elucidating the neurobiological underpinnings of SSDs, particularly central sensitization, autonomic dysfunction, and dysregulated gut-brain and neuroimmune interactions that perpetuate symptom amplification (66). Advances in functional neuroimaging, computational psychiatry, and neuroendocrinology offer promising pathways for refining diagnostic criteria and identifying objective biomarkers, paving the way for precision medicine approaches in managing SSDs (67). By integrating emerging insights from neuroscience, psychoneuroimmunology, and psychosomatic medicine, a more holistic and biologically informed model of SSDs can be developed one that moves beyond symptom management toward targeted, mechanistically driven, and personalized interventions (68).

#### Adequacy of assessment

Clarifying the mind-body interaction requires comprehensive psychiatric and medical assessments. Clinical observation and individualized judgment are foundational to medicine, as emphasized by Sir William Osler: “There is no more difficult art to acquire than the art of observation.” Medicine should prioritize treating patients over diseases, adapting to everyone’s needs rather than relying solely on population-based guidelines (49). Guidelines should include disclaimers acknowledging their limitations and emphasizing the role of clinical judgment. Patient experience should be valued equally with scientific evidence, as noted by the UK National Health Service: “Patient experience evidence should be respected, cherished, and used on an equal footing with medical evidence (83).

#### Toward precision psychiatry: integrating biomarkers, AI, and neuroadaptive therapies

The evolving understanding of somatic symptom disorders (SSDs) and related conditions necessitates a paradigm shift in diagnostic and treatment approaches, moving beyond traditional symptom-based classifications toward an integrative, biomarker-driven, and computationally informed framework (82). Historically, conditions such as fibromyalgia, chronic fatigue syndrome, and post-viral syndromes were dismissed as psychogenic due to the absence of identifiable structural abnormalities, yet emerging evidence implicates immune dysregulation, neuroinflammation, and autonomic dysfunction in their pathophysiology, emphasizing the need for revised diagnostic criteria that incorporate neurological and systemic biomarkers (84). The reinterpretation of SSDs challenges outdated clinical biases, advocating for a probabilistic, systems-based approach that evaluates symptoms within the broader network of neurobiological, metabolic, and immunological interactions. This shift also extends to treatment, where reliance on symptom suppression and psychiatric interventions must evolve into precision medicine strategies integrating neurobiologically informed protocols, multimodal psychiatric interventions, and pharmacogenomic-driven approaches tailored to individual neuroimmune and genetic profiles (85). Advances in computational psychiatry are redefining diagnostics through AI-driven biomarkers, leveraging machine learning to subtype symptoms, predict treatment responses, and enhance diagnostic accuracy using neuroimaging, EEG, and neuroinflammatory markers (86). Wearable biosensors and smartphone-based digital phenotyping further enable real-time monitoring of autonomic reactivity, cognitive-affective states, and sensorimotor symptoms, bridging the gap between clinical assessments and continuous physiological tracking (87). In parallel, integrating neurodiagnostic algorithms with inflammatory, autonomic, and neurophysiological biomarkers enhances differentiation between psychosomatic, neuroimmune, and functional disorders, paving the way for personalized interventions (88). Emerging neuroadaptive therapies, including vagus nerve stimulation, transcranial magnetic stimulation, and closed-loop neuromodulation, offer promising avenues for recalibrating autonomic function and sensory integration deficits, while AI-assisted psychotherapy personalizes cognitive-behavioral strategies using real-time biometric feedback (89, 90). This paradigm shift underscores the necessity for transdisciplinary collaboration, bridging neuroimmunology, computational neuroscience, and psychosomatic medicine to refine diagnostic frameworks and therapeutic interventions. The future of psychosomatic medicine lies in biomarker-guided, AI-assisted diagnostics, integrating digital health tools and neuroadaptive interventions to enhance real-time patient monitoring, optimize treatment strategies, and ultimately foster a more precise and biologically informed approach to brain-body disorders (91, 92).

## Conclusions: advancing understanding in the mind-body interface

Historically, unexplained physical symptoms were often attributed to psychiatric origins. This tendency has led to the misdiagnosis of many patients with complex, poorly understood illnesses who received inadequate evaluations. Limited integration between psychiatry and general medicine, restrictive diagnostic criteria, and flawed guidelines continue to contribute to diagnostic errors. Conditions like Lyme disease and ME/CFS are frequently misattributed to psychogenic causes. However, advances in technologies such as brain imaging and neurobiological assessments are increasingly revealing the pathophysiology underlying these conditions, challenging outdated concepts like psychogenic and functional disorders (16).

Accurate diagnosis requires clearly defined signs and symptoms consistent with established diagnostic criteria. A comprehensive clinical examination, including medical history, systems review, psychiatric assessment, and clinical judgment, is more reliable than relying solely on diagnostic tests. The absence of diagnostic findings should not automatically imply a psychiatric condition. Instead, the diagnosis of psychosomatic conditions requires a psychodynamic explanation, not exclusion due to the inability to identify another cause. Furthermore, the presence of a psychiatric diagnosis does not preclude the possibility of coexisting or causative somatic conditions (93).

Recognizing the inherent medical uncertainty in all diagnoses is crucial. Complex diseases require nuanced explanations, and the ethical approach involves continuous exploration of symptoms and their causes. Outdated terms such as medically unexplained symptoms, somatoform disorder, and compensation neurosis should be retired (49, 94). Subjective labels like vague or nonspecific symptoms are often misapplied. Additionally, over-diagnosis of conversion disorders, functional disorders, psychogenic conditions, and related terms can lead to inadequate treatment and mismanagement.

To advance diagnostic accuracy, it is essential to foster better education at the intersection of medicine and psychiatry, promote comprehensive training in the mind-body interface, and employ clinical judgment rooted in thorough assessment. To advance diagnostic accuracy, it is essential to foster better education at the intersection of medicine and psychiatry, promote comprehensive training in the mind-body interface, and employ clinical judgment rooted in thorough assessment (30, 31). Recognizing the limits of current knowledge and maintaining humility is fundamental to improving patient care and driving progress in both science and medicine.

## Data Availability

The original contributions presented in the study are included in the article/**Supplementary Material**, further inquiries can be directed to the corresponding author/s.

## References

[1] Bradford A Meyer AND Khan S Giardina TD Singh H . Diagnostic error in mental health: a review. BMJ Qual Saf. (2024) 33:663–72. doi: 10.1136/bmjqs-2023-016996 38575311 PMC11503128

[2] Overmier JB Murison R . Restoring Psychology’s Role in Peptic Ulcer. Appl Psychol Health Well Being. (2013) 5(1):5–27. doi: 10.1111/j.1758-0854.2012.01076.x 23457084 PMC3613748

[3] Plug I van Dulmen S Stommel W Olde Hartman TC Das E . Physicians’ and patients’ Interruptions in clinical practice: A quantitative analysis. Ann Fam Med. (2022) 20:423–9. doi: 10.1370/afm.2846 36228066 PMC9512556

[4] Andreassen OA Hindley GFL Frei O Smeland OB . New insights from the last decade of research in psychiatric genetics: discoveries, challenges and clinical implications. World Psychiatry. (2023) 22:4–24. doi: 10.1002/wps.21034 36640404 PMC9840515

[5] Deaton A Cartwright N . Understanding and misunderstanding randomized controlled trials. Soc Sci Med. (2018) 210:2–21. doi: 10.1016/j.socscimed.2017.12.005 29331519 PMC6019115

[6] Bobe JR Jutras BL Horn EJ Embers ME Bailey A Moritz RL . Recent progress in lyme disease and remaining challenges. Front Med (Lausanne). (2021) 8:666554. doi: 10.3389/fmed.2021.666554 34485323 PMC8416313

[7] Goetz LH Schork NJ . Personalized medicine: motivation, challenges, and progress. Fertil Steril. (2018) 109:952–63. doi: 10.1016/j.fertnstert.2018.05.006 29935653 PMC6366451

[8] Fagen JL Shelton JA Luché-Thayer J . Medical gaslighting and lyme disease: the patient experience. Healthcare (Basel). (2023) 12. doi: 10.3390/healthcare12010078 38200984 PMC10778834

[9] Wagenschieber E Blunck D . Impact of reimbursement systems on patient care - a systematic review of systematic reviews. Health Econ Rev. (2024) 14:22. doi: 10.1186/s13561-024-00487-6 38492098 PMC10944612

[10] Azubuike CD Alawode OA . Delayed healthcare due to cost among adults with multimorbidity in the United States. Healthcare (Basel). (2024) 12. doi: 10.3390/healthcare12222271 39595468 PMC11593596

[11] Meraya AM Raval AD Sambamoorthi U . Chronic condition combinations and health care expenditures and out-of-pocket spending burden among adults, Medical Expenditure Panel Survey, 2009 and 2011. Prev Chronic Dis. (2015) 12:E12. doi: 10.5888/pcd12.140388 25633487 PMC4310713

[12] Claréus B Renström EA . Physicians’ gender bias in the diagnostic assessment of medically unexplained symptoms and its effect on patient-physician relations. Scand J Psychol. (2019) 60:338–47. doi: 10.1111/sjop.12545 31124165 PMC6851885

[13] Armentor JL . Living with a contested, stigmatized illness: experiences of managing relationships among women with fibromyalgia. Qual Health Res. (2017) 27:462–73. doi: 10.1177/1049732315620160 26667880

[14] Yao J Lv D Chen W . Multiple myeloma, misdiagnosed as somatic symptom disorder: A case report. Front Psychiatry. (2018) 9:557. doi: 10.3389/fpsyt.2018.00557 30429803 PMC6220088

[15] Okobi OE Ayo-Farai O Tran M Ibeneme C Ihezie CO Ezie OB . The impact of infectious diseases on psychiatric disorders: A systematic review. Cureus. (2024) 16:e66323. doi: 10.7759/cureus.66323 39238736 PMC11377121

[16] Graves BS Patel M Newgent H Parvathy G Nasri A Moxam J . Chronic fatigue syndrome: diagnosis, treatment, and future direction. Cureus. (2024) 16:e70616. doi: 10.7759/cureus.70616 39483544 PMC11526618

[17] Adler BL Chung T Rowe PC Aucott J . Dysautonomia following Lyme disease: a key component of post-treatment Lyme disease syndrome? Front Neurol. (2024) 15:1344862. doi: 10.3389/fneur.2024.1344862 38390594 PMC10883079

[18] Kienle GS Kiene H . Clinical judgement and the medical profession. J Eval Clin Pract. (2011) 17:621–7. doi: 10.1111/j.1365-2753.2010.01560.x 20973873 PMC3170707

[19] Gopal DP Chetty U O’Donnell P Gajria C Blackadder-Weinstein J . Implicit bias in healthcare: clinical practice, research and decision making. Future Healthc J. (2021) 8:40–8. doi: 10.7861/fhj.2020-0233 33791459 PMC8004354

[20] Deumer U-S Varesi A Floris V Savioli G Mantovani E López-Carrasco P . Myalgic encephalomyelitis/chronic fatigue syndrome (ME/CFS): an overview. J Clin Med. (2021) 10. doi: 10.3390/jcm10204786 34682909 PMC8538807

[21] Cullinan J Pheby DFH Araja D Berkis U Brenna E de Korwin J-D . Perceptions of european ME/CFS experts concerning knowledge and understanding of ME/CFS among primary care physicians in europe: A report from the european ME/CFS research network (EUROMENE). Medicina (Kaunas). (2021) 57. doi: 10.3390/medicina57030208 33652747 PMC7996783

[22] McManimen S McClellan D Stoothoff J Gleason K Jason LA . Dismissing chronic illness: A qualitative analysis of negative health care experiences. Health Care Women Int. (2019) 40:241–58. doi: 10.1080/07399332.2018.1521811 30829147 PMC6567989

[23] Cortes Rivera M Mastronardi C Silva-Aldana CT Arcos-Burgos M Lidbury BA . Myalgic encephalomyelitis/chronic fatigue syndrome: A comprehensive review. Diagnostics (Basel). (2019) 9. doi: 10.3390/diagnostics9030091 31394725 PMC6787585

[24] Vink M Vink-Niese A . The updated NICE guidance exposed the serious flaws in CBT and graded exercise therapy trials for ME/CFS. Healthcare (Basel). (2022) 10. doi: 10.3390/healthcare10050898 35628033 PMC9141828

[25] Johnson L Stricker RB . The Infectious Diseases Society of America Lyme guidelines: a cautionary tale about the development of clinical practice guidelines. Philos Ethics Humanit Med. (2010) 5:9. doi: 10.1186/1747-5341-5-9 20529367 PMC2901226

[26] Lantos PM Rumbaugh J Bockenstedt LK Falck-Ytter YT Aguero-Rosenfeld ME Auwaerter PG . Clinical practice guidelines by the infectious diseases society of america (IDSA), american academy of neurology (AAN), and american college of rheumatology (ACR): 2020 guidelines for the prevention, diagnosis, and treatment of lyme disease. Arthritis Care Res (Hoboken). (2021) 73:1–9. doi: 10.1002/acr.24495 33251700

[27] Shaneyfelt T . In guidelines we cannot trust. Arch Intern Med. (2012) 172:1633–4. doi: 10.1001/2013.jamainternmed.335 23089851

[28] Eminson DM . Medically unexplained symptoms in children and adolescents. Clin Psychol Rev. (2007) 27:855–71. doi: 10.1016/j.cpr.2007.07.007 17804131

[29] Geist R Weinstein M Walker L Campo JV . Medically unexplained symptoms in young people: The doctor’s dilemma. Paediatr Child Health. (2008) 13:487–91. doi: 10.1093/pch/13.6.487 PMC2532910 19436430

[30] Presseau J Byrne-Davis LMT Hotham S Lorencatto F Potthoff S Atkinson L . Enhancing the translation of health behaviour change research into practice: a selective conceptual review of the synergy between implementation science and health psychology. Health Psychol Rev. (2022) 16:22–49. doi: 10.1080/17437199.2020.1866638 33446062

[31] Epstein LH Bickel WK Czajkowski SM Paluch RA Moeyaert M Davidson KW . Single case designs for early phase behavioral translational research in health psychology. Health Psychol. (2021) 40:858–74. doi: 10.1037/hea0001055 34370494 PMC8738131

[32] Stein DJ Palk AC Kendler KS . What is a mental disorder? An exemplar-focused approach. Psychol Med. (2021) 51:894–901. doi: 10.1017/S0033291721001185 33843505 PMC8161428

[33] Galderisi S Heinz A Kastrup M Beezhold J Sartorius N . Toward a new definition of mental health. World Psychiatry. (2015) 14:231–3. doi: 10.1002/wps.20231 26043341 PMC4471980

[34] Bradley L Noble N Hendricks B . DSM-5-TR: salient changes. Family J. (2023) 31:5–10. doi: 10.1177/10664807221123558

[35] Goldberg D . Vulnerability factors for common mental illnesses. Br J Psychiatry. (2001) 178. doi: 10.1192/bjp.178.40.s69 11315228

[36] D’aes T Mariën P . Cognitive and affective disturbances following focal brainstem lesions: a review and report of three cases. Cerebellum. (2015) 14:317–40. doi: 10.1007/s12311-014-0626-8 25520275

[37] Marvel CL Paradiso S . Cognitive and neurological impairment in mood disorders. Psychiatr Clin North Am. (2004) 27:19–36. doi: 10.1016/S0193-953X(03)00106-0 15062628 PMC2570029

[38] Kirkbride JB Anglin DM Colman I Dykxhoorn J Jones PB Patalay P . The social determinants of mental health and disorder: evidence, prevention and recommendations. World Psychiatry. (2024) 23:58–90. doi: 10.1002/wps.21160 38214615 PMC10786006

[39] Eisma MC . Prolonged grief disorder in ICD-11 and DSM-5-TR: Challenges and controversies. Aust N Z J Psychiatry. (2023) 57:944–51. doi: 10.1177/00048674231154206 36748103 PMC10291380

[40] Prentice JC Pizer SD . Delayed access to health care and mortality. Health Serv Res. (2007) 42:644–62. doi: 10.1111/j.1475-6773.2006.00626.x 17362211 PMC1955366

[41] Hollingsworth A . The beginning of wisdom is the definition of terms” - socrates. Breast J. (2015) 21:119–20. doi: 10.1111/tbj.12370 25735206

[42] Nielsen K Ward T . Towards a new conceptual framework for psychopathology: Embodiment, enactivism, and embedment. Theory Psychol. (2018) 28:800–22. doi: 10.1177/0959354318808394

[43] Gaebel W Stricker J Kerst A . Changes from ICD-10 to ICD-11 and future directions in psychiatric classification. Dialogues Clin Neurosci. (2020) 22:7–15. doi: 10.31887/DCNS.2020.22.1/wgaebel 32699501 PMC7365296

[44] First MB Yousif LH Clarke DE Wang PS Gogtay N Appelbaum PS . DSM-5-TR: overview of what’s new and what’s changed. World Psychiatry. (2022) 21:218–9. doi: 10.1002/wps.20989 35524596 PMC9077590

[45] Shorter E . The history of nosology and the rise of the Diagnostic and Statistical Manual of Mental Disorders. Dialogues Clin Neurosci. (2015) 17:59–67. doi: 10.31887/DCNS.2015.17.1/eshorter 25987864 PMC4421901

[46] Stein DJ Shoptaw SJ Vigo DV Lund C Cuijpers P Bantjes J . Psychiatric diagnosis and treatment in the 21st century: paradigm shifts versus incremental integration. World Psychiatry. (2022) 21:393–414. doi: 10.1002/wps.20998 36073709 PMC9453916

[47] Scala JJ Ganz AB Snyder MP . Precision medicine approaches to mental health care. Physiol (Bethesda). (2023) 38. doi: 10.1152/physiol.00013.2022 36099270 PMC9870582

[48] Dalgleish T Black M Johnston D Bevan A . Transdiagnostic approaches to mental health problems: Current status and future directions. J Consult Clin Psychol. (2020) 88:179–95. doi: 10.1037/ccp0000482 32068421 PMC7027356

[49] Bransfield RC Friedman KJ . Differentiating psychosomatic, somatopsychic, multisystem illnesses, and medical uncertainty. Healthcare (Basel). (2019) 7. doi: 10.3390/healthcare7040114 31597359 PMC6955780

[50] Tatayeva R Ossadchaya E Sarculova S Sembayeva Z Koigeldinova S . Psychosomatic aspects of the development of comorbid pathology: A review. Med J Islam Repub Iran. (2022) 36:152. doi: 10.47176/mjiri.36.152 36636258 PMC9826781

[51] Huang K-Y Wang F-Y Lv M Ma X-X Tang X-D Lv L . Irritable bowel syndrome: Epidemiology, overlap disorders, pathophysiology and treatment. World J Gastroenterol. (2023) 29:4120–35. doi: 10.3748/wjg.v29.i26.4120 37475846 PMC10354571

[52] Qin H-Y Cheng C-W Tang X-D Bian Z-X . Impact of psychological stress on irritable bowel syndrome. World J Gastroenterol. (2014) 20:14126–31. doi: 10.3748/wjg.v20.i39.14126 25339801 PMC4202343

[53] Goldstein DS . Stress and the “extended” autonomic system. Auton Neurosci. (2021) 236:102889. doi: 10.1016/j.autneu.2021.102889 34656967 PMC10699409

[54] Schiele MA Gottschalk MG Domschke K . The applied implications of epigenetics in anxiety, affective and stress-related disorders - A review and synthesis on psychosocial stress, psychotherapy and prevention. Clin Psychol Rev. (2020) 77. doi: 10.1016/j.cpr.2020.101830 32163803

[55] Dar T Radfar A Abohashem S Pitman RK Tawakol A Osborne MT . Psychosocial stress and cardiovascular disease. Curr Treat Options Cardiovasc Med. (2019) 21:23. doi: 10.1007/s11936-019-0724-5 31028483 PMC6568256

[56] James KA Stromin JI Steenkamp N Combrinck MI . Understanding the relationships between physiological and psychosocial stress, cortisol and cognition. Front Endocrinol (Lausanne). (2023) 14:1085950. doi: 10.3389/fendo.2023.1085950 36950689 PMC10025564

[57] Medina-Leyte DJ Zepeda-García O Domínguez-Pérez M González-Garrido A Villarreal-Molina T Jacobo-Albavera L . Endothelial dysfunction, inflammation and coronary artery disease: potential biomarkers and promising therapeutical approaches. Int J Mol Sci. (2021) 22. doi: 10.3390/ijms22083850 33917744 PMC8068178

[58] Gros M Gros B Mesonero JE Latorre E . Neurotransmitter dysfunction in irritable bowel syndrome: emerging approaches for management. J Clin Med. (2021) 10. doi: 10.3390/jcm10153429 34362210 PMC8347293

[59] Pasta A Formisano E Calabrese F Plaz Torres MC Bodini G Marabotto E . Food intolerances, food allergies and IBS: lights and shadows. Nutrients. (2024) 16. doi: 10.3390/nu16020265 38257158 PMC10821155

[60] Taylor AG Goehler LE Galper DI Innes KE Bourguignon C . Top-down and bottom-up mechanisms in mind-body medicine: development of an integrative framework for psychophysiological research. Explore (NY). (2010) 6:29–41. doi: 10.1016/j.explore.2009.10.004 20129310 PMC2818254

[61] Gomez R Chen W Houghton S . Differences between DSM-5-TR and ICD-11 revisions of attention deficit/hyperactivity disorder: A commentary on implications and opportunities. World J Psychiatry. (2023) 13:138–43. doi: 10.5498/wjp.v13.i5.138 37303925 PMC10251354

[62] Wright SN Anticevic A . Generative AI for precision neuroimaging biomarker development in psychiatry. Psychiatry Res. (2024) 339:115955. doi: 10.1016/j.psychres.2024.115955 38909415 PMC11321914

[63] Hanna MG Pantanowitz L Dash R Harrison JH Deebajah M Pantanowitz J . Future of artificial intelligence—Machine learning trends in pathology and medicine. Modern Pathol. (2025) 38. doi: 10.1016/j.modpat.2025.100705 39761872

[64] Javed H El-Sappagh S Abuhmed T . Robustness in deep learning models for medical diagnostics: security and adversarial challenges towards robust AI applications. Artif Intell Rev. (2025) 58. doi: 10.1007/s10462-024-11005-930311153

[65] Allam S . Advancing patient-centered care: A nationwide analysis of hospital efficiency and morbidity using innovative propensity score techniques. Cureus. (2024) 16:e76370. doi: 10.7759/cureus.76370 39722658 PMC11669325

[66] Krystal JH State MW . Psychiatric disorders: diagnosis to therapy. Cell. (2014) 157:201–14. doi: 10.1016/j.cell.2014.02.042 24679536 PMC4104191

[67] Perez DL Nicholson TR Asadi-Pooya AA Bègue I Butler M Carson AJ . Neuroimaging in functional neurological disorder: state of the field and research agenda. NeuroImage Clin. (2021) 30:102623. doi: 10.1016/j.nicl.2021.102623 34215138 PMC8111317

[68] Mavroudis I Kazis D Kamal FZ Gurzu I-L Ciobica A Pădurariu M . Understanding functional neurological disorder: recent insights and diagnostic challenges. Int J Mol Sci. (2024) 25. doi: 10.3390/ijms25084470 38674056 PMC11050230

[69] Chen X Ai C Liu Z Wang G . Neuroimaging studies of resting-state functional magnetic resonance imaging in eating disorders. BMC Med Imaging. (2024) 24:265. doi: 10.1186/s12880-024-01432-z 39375605 PMC11460144

[70] Agarwal N Renshaw PF . Proton MR spectroscopy-detectable major neurotransmitters of the brain: biology and possible clinical applications. AJNR Am J Neuroradiol. (2012) 33:595–602. doi: 10.3174/ajnr.A2587 22207303 PMC4627491

[71] Vink M Vink-Niese A . CBT and graded exercise therapy studies have proven that ME/CFS and long COVID are physical diseases, yet no one is aware of that. Front Hum Neurosci. (2025) 19:1495050. doi: 10.3389/fnhum.2025.1495050 39944089 PMC11814198

[72] Claréus B Renström EA . Physicians’ gender bias in the diagnostic assessment of medically unexplained symptoms and its effect on patient–physician relations. Scand J Psychol. (2019) 60:338–47. doi: 10.1111/sjop.12545 31124165 PMC6851885

[73] Husain M Chalder T . Medically unexplained symptoms: assessment and management. Clin Med (Lond). (2021) 21:13–8. doi: 10.7861/clinmed.2020-0947 33479063 PMC7850206

[74] Spurrier GF Shulman K Dibich S Benoit L Duckworth K Martin A . Physical symptoms as psychiatric manifestations in medical spaces: A qualitative study. Front Psychiatry. (2022) 13:1074424. doi: 10.3389/fpsyt.2022.1074424 36683974 PMC9845882

[75] Smakowski A Hüsing P Völcker S Löwe B Rosmalen JGM Shedden-Mora M . Psychological risk factors of somatic symptom disorder: A systematic review and meta-analysis of cross-sectional and longitudinal studies. J Psychosom Res. (2024) 181:111608. doi: 10.1016/j.jpsychores.2024.111608 38365462

[76] Harvey SB Wessely S Kuh D Hotopf M . The relationship between fatigue and psychiatric disorders: evidence for the concept of neurasthenia. J Psychosom Res. (2009) 66:445–54. doi: 10.1016/j.jpsychores.2008.12.007 19379961 PMC3500687

[77] Targum SD Fava M . Fatigue as a residual symptom of depression. Innov Clin Neurosci. (2011) 8:40–3. PMC3225130 22132370

[78] Fink P Toft T Hansen MS Ørnbøl E Olesen F . Symptoms and syndromes of bodily distress: an exploratory study of 978 internal medical, neurological, and primary care patients. Psychosom Med. (2007) 69:30–9. doi: 10.1097/PSY.0b013e31802e46eb 17244846

[79] Scognamiglio P Iniziato V La Pia S Martiadis V . A no-win situation: psychiatrists navigating competing obligations between free will, paternalism, duty of care, and position of guarantee. Ment Wellness. (2023) 1. doi: 10.4081/mw.2023.4

[80] Morena D Di Fazio N Scognamiglio P Delogu G Baldari B Cipolloni L . Predictors of defensive practices among italian psychiatrists: additional findings from a national survey. Medicina (B Aires). (2023) 59:1928. doi: 10.3390/medicina59111928 38003977 PMC10673589

[81] Scognamiglio P Morena D Di Fazio N Delogu G Iniziato V La Pia S . Vox clamantis in deserto: a survey among Italian psychiatrists on defensive medicine and professional liability. Front Psychiatry. (2023) 14:1244101. doi: 10.3389/fpsyt.2023.1244101 37663598 PMC10469623

[82] Löwe B Levenson J Depping M Hüsing P Kohlmann S Lehmann M . Somatic symptom disorder: A scoping review on the empirical evidence of a new diagnosis. J Psychosom Res. (2022) 157:110876. doi: 10.1016/j.jpsychores.2022.110876 34776017 PMC8961337

[83] Usher-Smith JA Harte E MacLure C Martin A Saunders CL Meads C . Patient experience of NHS health checks: a systematic review and qualitative synthesis. BMJ Open. (2017) 7:e017169. doi: 10.1136/bmjopen-2017-017169 28801437 PMC5724113

[84] Komaroff AL Lipkin WI . ME/CFS and Long COVID share similar symptoms and biological abnormalities: road map to the literature. Front Med (Lausanne). (2023) 10:1187163. doi: 10.3389/fmed.2023.1187163 37342500 PMC10278546

[85] Dhieb D Bastaki K . Pharmaco-multiomics: A new frontier in precision psychiatry. Int J Mol Sci. (2025) 26:1082. doi: 10.3390/ijms26031082 39940850 PMC11816785

[86] Baydili İ Tasci B Tasci G . Artificial intelligence in psychiatry: A review of biological and behavioral data analyses. Diagnostics. (2025) 15:434. doi: 10.3390/diagnostics15040434 40002587 PMC11854694

[87] Madrid RE Ramallo A Barraza F; Chaile DE; Madrid RE Ramallo FA . Citation: smartphone-based biosensor devices for healthcare: technologies, trends, and adoption by end-users. Bioengineering (2022) 9. doi: 10.3390/bioengineering PMC8945789 35324790

[88] Goldsmith DR Bekhbat M Mehta ND Felger JC . Inflammation-related functional and structural dysconnectivity as a pathway to psychopathology. Biol Psychiatry. (2023) 93:405–18. doi: 10.1016/j.biopsych.2022.11.003 36725140 PMC9895884

[89] Vlaicu A BustuChina Vlaicu M . New neuromodulation techniques for treatment resistant depression. Int J Psychiatry Clin Pract. (2020) 24:106–15. doi: 10.1080/13651501.2020.1728340 32069166

[90] Austelle CW Cox SS Wills KE Badran BW . Vagus nerve stimulation (VNS): recent advances and future directions. Clin Auton Res. (2024) 34:529–47. doi: 10.1007/s10286-024-01065-w 39363044 PMC11543756

[91] Levkovich I . Is artificial intelligence the next co-pilot for primary care in diagnosing and recommending treatments for depression? Med Sci (Basel). (2025) 13. doi: 10.3390/medsci13010008 39846703 PMC11755475

[92] Farmaki A Manolopoulos E Natsiavas P . Will precision medicine meet digital health? A systematic review of pharmacogenomics clinical decision support systems used in clinical practice. Omics. (2024) 28:442–60. doi: 10.1089/omi.2024.0131 39136110

[93] Efremov A . Psychosomatics: communication of the central nervous system through connection to tissues, organs, and cells. Clin Psychopharmacol Neurosci. (2024) 22:565–77. doi: 10.9758/cpn.24.1197 39420604 PMC11494424

[94] Marks EM Hunter MS . Medically Unexplained Symptoms: an acceptable term? Br J Pain. (2015) 9:109–14. doi: 10.1177/2049463714535372 26516565 PMC4616968

