# Correction: Case Report: The intersection of psychiatry and medicine: diagnostic and ethical insights from case studies

**DOI:** 10.3389/fpsyt.2026.1992650

**Published:** 2026-10-06

**Authors:** Francesco Monaco, Annarita Vignapiano, Martina D’Angelo, Fabiola Raffone, Valeria Di Stefano, Antonella Boccia, Anna Longobardi, Benedetta Di Gruttola, Michele Fornaro, Giulio Corrivetti, Iolanda Martino, Luca Steardo, Luca Steardo Jr

**Affiliations:** 1 Department of Mental Health, Azienda Sanitaria Locale (ASL) Salerno, Salerno, Italy; 2 European Biomedical Research Institute of Salerno (EBRIS), Salerno, Italy; 3 Psychiatric Unit, Department of Health Sciences, University Magna Graecia of Catanzaro, Catanzaro, Italy; 4 Department of Mental Health, Azienda Sanitaria Locale (ASL) Napoli 1 Centro, Napoli, Italy; 5 Section of Psychiatry, Department of Neuroscience, Reproductive Science, and Dentistry, Federico II University of Naples, Naples, Italy; 6 Department of Medical Sciences, Institute of Neurology, University “Magna Graecia” of Catanzaro, Catanzaro, Italy; 7 Department of Physiology and Pharmacology “Vittorio Erspamer”, Sapienza University of Rome, Rome, Italy; 8 Department of Clinical Psychology, University Giustino Fortunato, Benevento, Italy

**Keywords:** psychosomatic disorders, somatopsychic conditions, complex systemic disorders, diagnostic challenges, medicine-psychiatry interface, ethical implications

In the original article, there was an error in the reference supporting the statement concerning the historical attribution of poorly understood illnesses to psychiatric causes. The refernece was erroneously written as:

“*De Hert M, Correll CU, Bobes J, Cetkovich-Bakmas M, Cohen D, Asai I*, et al. *Physical illness in patients with severe mental disorders. I. Prevalence, impact of medications and disparities in health care. World Psychiatry. 2011;10(1):52–77. doi:10.1002/j.2051-5545.2011.tb00014.x*”

The correct reference is:

“*Overmier JB, Murison R. Restoring Psychology’s Role in Peptic Ulcer. Appl Psychol Health Well Being. 2013;5(1):5–27. doi:10.1111/j.1758-0854.2012.01076.x*”

This review describes the historical attribution of peptic ulcer disease primarily to psychological factors and the subsequent shift toward an infectious model following the recognition of the role of Helicobacter pylori.

A correction has been made to the **Introduction**, paragraph 1. The original sentence stated:

“Historically, poorly understood illnesses have frequently been attributed to psychiatric causes until their pathophysiology was better elucidated (2).”

The sentence has now been revised to:

“Historically, poorly understood illnesses have frequently been attributed to psychiatric causes until their pathophysiology was better elucidated, as exemplified by the historical attribution of peptic ulcer disease primarily to psychological factors prior to the recognition of Helicobacter as one of its principal causes, alongside NSAID use (2).”

*The original version of this article has been updated.*

