# Supplementary material for: Case Report: The intersection of psychiatry and medicine: diagnostic and ethical insights from case studies
Source: Front Psychiatry. 2025 Apr 22;16:1576179. doi: 10.3389/fpsyt.2025.1576179 (PMC12053010; doi:10.3389/fpsyt.2025.1576179)
Supplement: Supplementary file 1 [file Table1.docx]

**Case Summary: Diagnostic and Therapeutic Insights at the Psychiatry-Medicine Interface**

| **Case** | **Main Symptoms** | **Differential Diagnosis** | **Diagnostic Tests** | **Therapeutic Outcome** |
| --- | --- | --- | --- | --- |
| Anorexia Nervosa (Binge/Purge) & Borderline Personality Disorder | Severe dietary restriction, binge eating, self-induced vomiting, seizure-like episodes, emotional instability | Epilepsy, syncope, major depressive disorder | EEG, ECG, psychometric assessments (EDE-Q, EDI-3) | Multidisciplinary treatment, partial recovery, reduced emergency visits |
| Anorexia Nervosa with Prominent Somatic Symptoms | Severe fatigue, concentration difficulties, somatic complaints, intermittent agitation episodes | Chronic fatigue syndrome, autoimmune disorder, major depressive disorder | SCL-90, PHQ-15, MMPI-2, metabolic blood tests | Gradual improvement with psychotherapy, pharmacotherapy, symptom management |
| Anorexia Nervosa & Obsessive-Compulsive Disorder | Weight loss, compulsive food-related behaviors, obsessive thoughts, social anxiety | Generalized anxiety disorder, autism spectrum disorder, bipolar disorder | OCI-R, CIA, EDE-Q, structured psychiatric interview | Behavioral therapy and SSRI treatment led to symptom stabilization |
| Functional Neurological Disorder (FND) with Sensorimotor Symptoms | Episodic limb weakness, gait disturbances, non-epileptic seizures, dissociative episodes | Multiple sclerosis, stroke, autoimmune neuropathy, myopathy | MRI, EEG, lumbar puncture, Hoover’s sign, electrophysiological studies | CBT, graded physical rehabilitation, psychiatric follow-up; functional improvement |
| Severe Functional Gastrointestinal Disorder & Somatic Symptom Disorder | Persistent abdominal pain, nausea, bloating, diarrhea/constipation, health-related anxiety | Inflammatory bowel disease, celiac disease, gut motility disorder | Endoscopy, stool tests, abdominal ultrasound, PHQ-15, HADS | Gut-directed hypnotherapy, dietary modifications, tricyclic antidepressants; partial symptom relief |

# **Clarification of Key Terminology Used in the Manuscript**

| **Term** | **Definition** | **Relevance in Manuscript** | **Nosological Reference** |
| --- | --- | --- | --- |
| Psychosomatic Disorders | Physical conditions that are caused or exacerbated by psychological factors such as chronic stress, anxiety, or emotional conflict. | Discussed in relation to historical misclassification of somatic symptoms and the stress-related worsening of certain medical conditions (e.g., IBS, cardiovascular disease). | Not formally defined in DSM-5-TR or ICD; used as a clinical concept. |
| Somatopsychic Conditions | Psychiatric symptoms or syndromes that arise from or are worsened by underlying medical or physiological conditions (e.g., endocrine disorders, autoimmune diseases, infections). | Used to highlight the inverse of psychosomatic disorders, emphasizing the need to rule out physical causes before assigning psychiatric diagnoses. | Not explicitly classified in DSM-5-TR or ICD; emerging in literature. |
| Functional Disorders | Conditions characterized by symptoms without structural abnormalities detectable by conventional tests, but with evidence of altered function (e.g., Functional Neurological Disorder, functional GI disorders). | Addressed through case examples (e.g., FND) and in critique of guidelines that underestimate brain-body dysfunction in these conditions. | Some recognized in DSM-5-TR (e.g., FND); ICD-11 includes functional categories. |
| Medically Unexplained Symptoms (MUS) | Physical symptoms that persist without clear medical explanation after appropriate investigation. Considered controversial and potentially stigmatizing. | Critically examined for its overuse in cases where diagnostic certainty is lacking. Emphasized as a label that should not preclude further investigation. | Removed from DSM-5-TR; previously used in clinical practice and older guidelines. |
